# Impact of periprocedural myocardial necrosis on short term clinical outcome

**DOI:** 10.1186/2197-425X-3-S1-A954

**Published:** 2015-10-01

**Authors:** M Hamdi, Y Hagag, M Khaled, A Elhadidy

**Affiliations:** Cairo University, Cairo, Egypt

**Keywords:** Cardiac biomarkers, PCI, MACENTRODUCTION

## Objectives

To assess the prognostic value of periprocedural myocardial injury during elective in the early post intervention period.

## Background

Elevations in the biochemical markers of myocardial damage are frequently detected after percutaneous coronary revascularization, but their clinical significance is still uncertain.

## Methods

CK, CK-MB, and cTn I were prospectively assessed in 100 patients who underwent elective PCI during the period between Jan 2012 and Jan 2013.

Patients were subjected to history taking, routine laboratory investigations, electrocardiogram ( ECG), cardiac biomarkers assay, and Echocardiography pre and post PCI.

The primary end point was death, myocardial infarction or severe, recurrent ischemia at 90 days.

## Results

Overall, 30 patients (30%) had elevated cardiac biomarkers after PCI. Elevated cardiac biomarkers were associated with significantly higher risks of the primary end point (MACE) in the early post intervention period (43.3% vs. 12.9%; p < 0.001) and a significantly higher risk of 3 months follow up MACE (66.7% vs. 14.3%; p < 0.001).

Elevation of cardiac biomarkers is more likely in older patients (p _ 0.034) , diabetics (96.7% vs. 11.4%; p < 0.001). , those with history of old MI(76.7% vs. 11.4%; p < 0.001). , and patients who had CHF (30% vs. 4.3%; p < 0.001) .while the gender , previous history of CVS ,COPD, and liver cirrhosis were non significant predictors of elevated cardiac biomarkers.

## Conclusions

Elevated cardiac biomarkers, often observed after elective PCI in patients with IHD, is associated with worse early post intervention and 90-day clinical outcomes. This marker, therefore, is a useful prognostic indicator in such patients.

